# Total Levels of Hippocampal Histone Acetylation Predict Normal Variability in Mouse Behavior

**DOI:** 10.1371/journal.pone.0094224

**Published:** 2014-05-02

**Authors:** Addie May I. Nesbitt, Richard D. McCurdy, Sharell M. Bryant, Mark D. Alter

**Affiliations:** 1 Center for Neurobiology and Behavior, Department of Psychiatry, University of Pennsylvania, Philadelphia, Pennsylvania, United States of America; 2 Department of Psychiatry and Pharmacology, University of Pennsylvania, Philadelphia, Pennsylvania, United States of America; University of Insubria, Italy

## Abstract

**Background:**

Genetic, pharmacological, and environmental interventions that alter total levels of histone acetylation in specific brain regions can modulate behaviors and treatment responses. Efforts have been made to identify specific genes that are affected by alterations in total histone acetylation and to propose that such gene specific modulation could explain the effects of total histone acetylation levels on behavior — the implication being that under naturalistic conditions variability in histone acetylation occurs primarily around the promoters of specific genes.

**Methods/Results:**

Here we challenge this hypothesis by demonstrating with a novel flow cytometry based technique that normal variability in open field exploration, a hippocampus-related behavior, was associated with total levels of histone acetylation in the hippocampus but not in other brain regions.

**Conclusions:**

Results suggest that modulation of total levels of histone acetylation may play a role in regulating biological processes. We speculate in the discussion that endogenous regulation of total levels of histone acetylation may be a mechanism through which organisms regulate cellular plasticity. Flow cytometry provides a useful approach to measure total levels of histone acetylation at the single cell level. Relating such information to behavioral measures and treatment responses could inform drug delivery strategies to target histone deacetylase inhibitors and other chromatin modulators to places where they may be of benefit while avoiding areas where correction is not needed and could be harmful.

## Introduction

Chromatin organization is a tightly regulated process important for gene expression regulation[1], [2]. Genes with higher levels of histone acetylation (AcH), a modification to histone tails that opens chromatin and increases accessibility to transcriptional machinery, are generally associated with higher levels of expression[3]. Suggesting an important role for regulation of AcH in biological processes, levels of AcH around the promoters of the *Glial cell-derived neurotrophic factor* (*Gdnf*) and *glucocorticoid receptor* (*NR3C1*) genes were associated with stress resilience and levels of maternal care, respectively[4], [5]. With respect to maternal care, rodent pups exposed to low levels of maternal care exhibited behavioral changes in adulthood that were reversed following treatment with histone deacetylase inhibitors (HDACi), compounds that increase total levels of AcH throughout the genome[6], [7]. However, it was unclear from these studies whether modulation of total AcH acted as a blunt intervention which elicited biologically important specific effects at certain genes and non-specific effects at many others, or whether regulation of total AcH is an important biological process in itself. Documented differences in AcH levels at specific gene promoters have been interpreted as support for the former, however, other studies lend support to the latter. For instance, memory-inducing stimuli[8], [9] and cocaine administration[10] were both reported to elicit changes in total AcH levels in the hippocampus and striatum, respectively. In these cases, specific genes were also identified that had significant changes in AcH around their promoters in response to stimuli, however, changes in AcH levels at these limited promoters could not account for the full extent of shifts in total AcH across the genome.

Regulation of total levels of histone acetylation may also play a role in antidepressant responses. For instance, chronic impramine, a tricyclic antidepressant medication, decreased expression levels of the *histone deacetylase 5* gene (*HDAC5*) leading to increases in total acetylated histone H3 (AcH3)[11]. Over-expression of *HDAC5* in the dentate gyrus region of the hippocampus blocked the behavioral effects of imipramine in a mouse model of depression[11]. However, it is currently unknown whether normal variability in behavior is related to variability in total levels of AcH in the absence of specific interventions. Supporting this possibility, preliminary experiments found that total levels of histone H3 acetylation in the hippocampus of Balb c/J mice, an anxious strain with high levels of stable inter-individual variability in anxiety-like behavior[12], were significantly associated with levels of anxiety-like behavior in the open-field test. To confirm these results and to potentially generalize a relationship of open-field behaviors to levels of histone H3 acetyaltion in other brain regions, we evaluated levels of total histone acetylation in several brain regions including the hippocampus in a new cohort of Balb c/J mice.

Since our assay required measurement of total AcH across a large number of samples, we developed a high-throughput method of analysis by modifying a flow cytometry protocol previously established to measure adult hippocampal neurogenesis[13]. By combining flow cytometric detection of AcH with behavioral testing, we found that individual levels of open field exploratory behavior were positively associated with levels of total histone H3 acetylation (AcH3) in the hippocampus, but not in other brain regions, of genetically identical mice. With regard to our method of AcH3 analysis, we found flow cytometry to be a reliable technique, providing a quick and detailed assessment of histone acetylation, capable of assaying more samples and requiring less input material than traditional Western blotting. Results suggest that previous studies in which total levels of histone acetylation were genetically or pharmacologically altered may mimic established physiological mechanisms important for brain development and phenotypic variability. An important implication of this work is that therapeutic strategies to target regulation of total levels of histone acetylation or other chromatin modifications at specific times, specific regions, and in specific cells within the brain may be more effective and naturalistic than strategies aimed at targeting chromatin modifications associated with specific genes.

## Materials and Methods

### Animals

Twenty adult female Balb c/J mice (stock number: 000651, Jackson Laboratories, Bar Harbor, ME) were used for the open-field experiments. Mice were group housed on a 12 hr light/dark cycle and provided food and water *ad libitum*. This study was carried out in strict accordance with the recommendations in the Guide for the Care and Use of Laboratory Animals of the National Institutes of Health. All animal protocols were reviewed and approved by the University of Pennsylvania IACUC board (Permit Number 801244).

### Behavioral Study

Mice were acclimated to the testing room for at least 30 minutes prior to open-field testing. Movements were captured by infrared beam breakage and recorded over a thirty-minute period and broken down into 6 intervals of 5 minutes using open field boxes (San Diego Instuments, San Diego, CA). Testing was conducted over a series of three days in either light or dark conditions: session 1 (day 1- light), session 2 (day 3– dark) and session 3 (day 28– light).

### Tissue collection and preparation

Immediately following euthanasia, brain tissue was placed in HBSS (Gibco) and gently minced for 30 sec. Five hundred uL of an enzyme solution (1 mg/mL papain, 250 units DNase and 0.1 M L-cystine in HBSS) was added and incubated at 37° for 15 mins to digest tissue and prevent cell adhesion. Immediately following enzymatic digestion, 500 uL of 1∶9 FBS:DMEM was added to halt enzymatic activity. Tissues were triturated by multiple passages through progressively smaller-bored pipettes to form a single cell suspension: P-1000, P-200 and a pasteur pipette (heat-treated to reduce bore size). Samples were then fixed in 4% paraformaldehyde for 10 mins. At this point, half of the sample was stored at 4° for repeat analysis. The remainder was permeabilized in 1% triton-X 100 for 10 mins and stained with 25 uL of 10 ug/mL diamidino-2-phenylindole (DAPI) for 10 mins. All washes were performed with 1% BSA/PBS. Cells were incubated overnight at 4° with 25 uL of 1∶24 PE-conjugated antibody against acetylated histone H3 (Millipore – FCABS325PE – rabbit anti-H3 acetyl K9,14). The following day, cells were washed, stained with of 10 mg/mL DAPI and washed again. Final resuspension in a solution of 2% BSA and 2 mM HEPES in PBS. Staining was analyzed with a BD Bioscience FACSCanto flow cytometer. A PE-congugated IgG Ab was used as a negative control for staining.

### Flow cytometry detection of H3 Acetylation

Detection of DAPI and PE levels were conducted on a BD FACSCanto. Data analysis was performed with FlowJo (Tree Star, Inc). The median intensity of PE fluorescence in DAPI-positive cells was used to split the mice into groups of high (upper 50%) and low (lower 50%) levels of PE staining.

### Western blot confirmation

Histones were acid extracted from the same brain tissue or cell culture samples used for flow cytometry. Next, histones were run on an Invitrogen Novex gel and stained with antibodies to both total and acetylated H3. Detection of secondary Abs and data analysis was performed using the Odyssey quantitative western blotting system. Primary antibodies were a rabbit polyclonal to AcH3 (Millipore: 17–615) and mouse monoclonal to total histone H3 (Abcam: mAbcam10799). Secondary antibodies were IRDye 680 donkey anti-mouse (Li-COR: 926–32222) and IRDye 800 donkey anti-rabbit (LI-COR 926–32213). The use of primary antibodies from different species allowed for staining and measurement of AcH3 and total H3 on the same sample at the same time. The level of AcH3 was calculated as the ratio of AcH3 staining divided by staining for total H3.

### Statistics

Statistical analysis was performed using StatView (SAS) and Prism 5 (Graphpad). Spearman correlations were used for correlations with behavior when data was not normally distributed.

## Results

### Detection of histone H3 acetylation by flow cytometry

**Figure 1:**
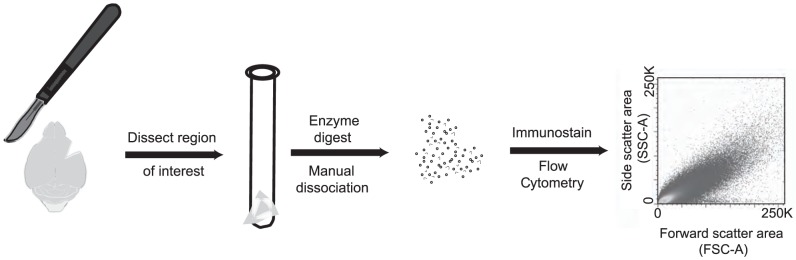
Schematic of flow cytometry protocol. Specific brain regions are microdisected, enzymatically digested, and dissociated via trituration. Subsequently, dissociated cells are immunostained with anti-AcH3 antibody, labeled with diamidino-2-phenylindole (DAPI) dye to identify stained nuclei, and run on a flow cytometer. Levels of AcH3 are measured as the median fluorescence intensity of DAPI-positive events.

Based on previous evidence within inbred mice linking large-scale changes in the hippocampal transcriptome to behavior in the open-field (OF) test[12], we decided to determine possible epigenetic correlates of OF behavior by examining total levels of histone acetylation (AcH) throughout the genome using flow cytometry. Early applications studying epigenetic modifications with flow included determining levels of AcH in cultured cells[14]. More recently, flow was used to measure total AcH levels in neurons, by looking at nuclei isolated from frozen brain tissue[15]. For our assay, we determined the levels of total histone H3 acetylation (AcH3) in fresh brain tissue by modifying a protocol previously established to measure adult hippocampal neurogenesis[13]. This technique involved single-cell separation via trituration[16], a process where cells are passed though small-bored pipettes to dissociate them, followed by immunostaining, and finally flow cytometry. In order to ensure that flow cytometry events were recorded from actual cells, we incorporated DAPI-based DNA staining to enhance detection of cell nuclei. Tissue was then processed as described (Methods) and analyzed on a flow cytometer (figure 1). The level of total AcH3 was indicated by the intensity of the antibody-conjugated fluorochrome, pychoerythrin (PE), in DAPI-positive cells. Because our assay was produced specifically for this study, we validated our method using an alternative method of protein quantification, Western blot. While both procedures require immuolabeling, Western blotting is performed on protein extracts, whereas flow cytometry is performed on cells. Samples from cell culture (figure S1a) or triturated brain (figure S1b) with high or low levels of AcH3 detected by flow were subsequently analyzed by Western blot. In both cultured cells and in triturated brain tissue, samples that had higher levels of AcH3 by flow cytometry also had increased levels of AcH3 by Western blot (figure S1). To confirm that flow cytometry measurements were technically reproducible, samples were evaluated with flow cytometry then stored overnight at 4 degrees Celsius and re-analyzed in a separate flow cytometry run. Results from separate runs were highly correlated indicating that measurement of AcH3 with flow cytometry was technically reproducible (figure S2 - Pearson R = 0.95, P<0.0001).

### Total levels of hippocampal histone H3 acetylation are related to levels of exploratory behavior

The open-field test involves a conflict between a natural drive of mice to explore with an innate fear of open spaces. We previously reported that individual differences in multiple aspects of open-field behavior in female Balb/c mice were stable over months, robust to changes in testing conditions, and associated with large-scale changes in gene expression levels [12]. Here we tested whether stable individual variability in open-field exploratory behavior was associated with variability in total levels of AcH3 using our flow cytometry technique.

Measures of open-field exploration of twenty Balb/c female mice were recorded during three 30-minute open-field sessions. Females were used for consistency with previous studies[12]. Importantly, previous studies indicated that behavioral differences were stable over multiple consecutive testing days spanning an entire menstrual cycle, indicating that any behavioral variability associated with the menstrual cycle was not sufficient to obscure the predominant effects of underlying non-genetic differences in levels of open-field exploration. Similarly in the current study, behavioral differences were also stable across all testing days (table S1).

**Figure 2:**
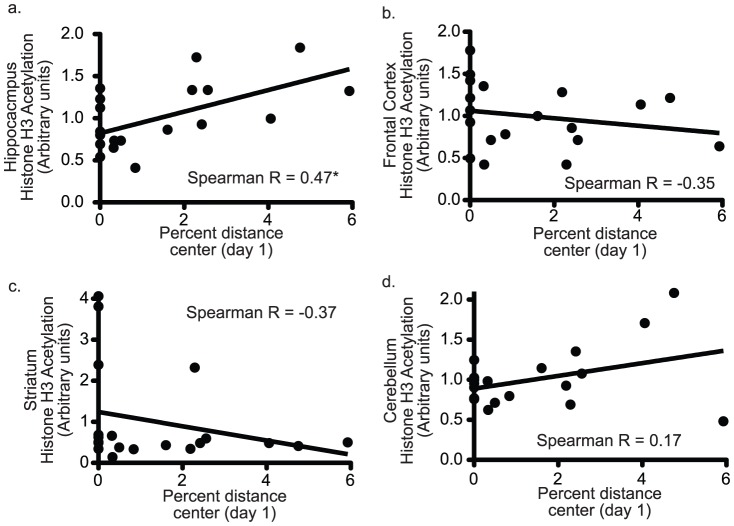
Levels of AcH3 in the hippocampus predict levels of open-field exploration. Figure shows bivariate plots of percent distance travelled in the center on day 1 (x-axis) versus AcH3 levels in various brain regions. There was a significant correlation between percent center and AcH3 in the hippocampus (panel a – Spearman r = 0.47*) but not for other regions (panels b–d). Spearman r correlation values for other open-field measures are found in table S1.

**Figure 3:**
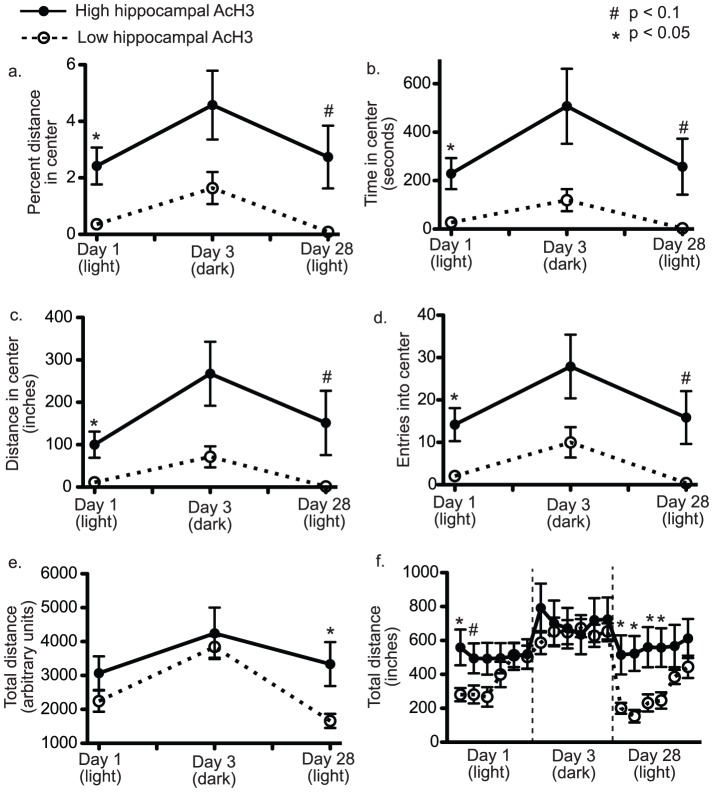
Levels of AcH3 in the hippocampus predict levels of anxiety-like behavior. Mice were split at the median by hippocampal AcH3. Groups of mice separated by hippocampal AcH3 were significantly different in nearly all center measures (Percent distance in center (a), Time in center (b), Distance in center (c), Entries into center (d)) on all testing days. Differences decreased for total distance travelled (panel e), especially when testing was done in the dark. Panel (f) shows a more detailed time course for Total distance by 5-minute bins. Figure shows that differences between groups were most pronounced at the beginning of sessions in the light, whereas, differences decreased in the dark and towards the end of sessions.

**Figure 4:**
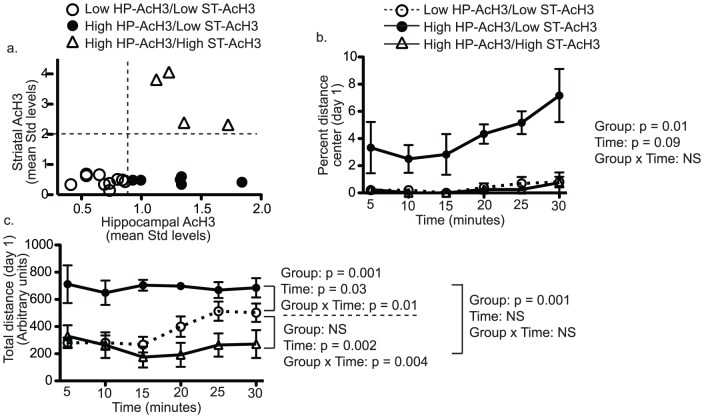
Possible interaction between AcH3 in the hippocampus and striatum. A group of four mice had high levels of AcH3 in the striatum and hippocampus (panel a). In contrast with mice that had high AcH3 only in the hippocampus, these mice were very hypoactive, and were even less active than low hippocampal AcH3 mice. Panel (b) shows a detailed time course for percent distance in the center on day 1 and demonstrates that in this measure, mice with high AcH3 in both regions were indistinguishable from low AcH3 mice (repeated measures ANOVA - main effect of group p = 0.01). Panel (c) shows a time course of total distance on day 1 and demonstrates that this group of mice was significantly less active than mice with high AcH3 only in the hippocampus (repeated measures ANOVA - main group effect p = 0.001) and mice with low levels of AcH3 in the hippocampus (repeated measures ANOVA - time x group interaction p = 0.004). Similar results were seen on other testing days (not shown).

Following behavioral testing, mice were returned to their home cages for four months to decrease the possibility that behavioral testing would contribute to levels of histone acetylation. At the time of sacrifice, tissue was obtained from the hippocampus, frontal cortex, cerebellum, and striatum. Acetylation of histone H3 (AcH3) was evaluated by flow cytometry. After comparing acetylation levels to behavior, we found total levels of AcH3 in the hippocampus, but not in other brain regions (figure 2b–d), were positively correlated with measures of open-field exploration (figure 2a – Spearman r = 0.47, p<0.05). Overall, higher levels of hippocampal AcH3 correlated with increases on day 1 in all open-field center measures (total time spent in center, total center distance traveled, number of entries into the center, and percent distance traveled in the center - table S2), but not in total distance traveled, suggesting levels of hippocampal AcH3 were related to anxiety-like behavior and not simply to hyperactivity. Despite the stability of behavioral differences across testing days, day-to-day relationships between open-field behaviors of individual mice are not always linear. This is because center measures in the open-field, which are suggested to measure anxiety-like behavior, can only discriminate between mice when mice enter the center. This confound was particularly evident on day 28 when only 40% of mice entered the center during the 30-minute test. Thus, we also found it useful to look for relationships between total levels of histone H3 acetylation and open-field behavior by dividing mice into groups based on a median split by AcH3 levels. Using this approach, it was noted that the group of mice with high levels of hippocampal AcH3 were significantly different from the low AcH3 group in nearly all open-field measures on day 1 with a trend for significance on day 3. Group analysis was also useful in suggesting that AcH3 may be more related to anxiety-like behavior than to general activity (figure 3). For instance, when each 30 minute session was separated into 5 minute bins, it was evident that differences in total distance travelled between high and low AcH3 groups were only present at the beginning of sessions in the light, whereas, differences disappeared at the end of sessions and in the testing session conducted in the dark (figure 4f). Because the beginning of sessions and testing in the light are more anxiogenic than the ends of sessions and testing in the dark, these results are consistent with AcH3 levels being more related to anxiety-like behavior than to general activity the (figure 3f). Of note, there was also a statistical association between striatal AcH3 and some open field measures (table S2). However, these effects were primarily related to four unusual mice (described below). When these mice were removed from the analysis the relationship of behavior with striatal AcH3 was no longer significant. Interestingly, because these mice also had high levels of hippocampal AcH3 and were very hypoactive, the relationship of increased center exploration with increased hippocampal AcH3 became even more significant when the four high striatal AcH3 mice were removed from the analysis (see below).

### Total levels of AcH3 in the striatum may interact with levels of hippocampal AcH3

For the most part, levels of AcH3 were not related across brain regions. However, there was a group of four mice that had high levels of AcH3 in the hippocampus and in the striatum (figure 4a). Upon closer examination, this group of mice behaved very differently from mice with high levels of AcH3 only in the hippocampus. Mice with increased AcH3 in both regions had a significantly lower percent distance travelled in the center on the first day of testing than mice with high levels of AcH3 only in the hippocampus (p = 0.01**) and were indistinguishable from mice with low levels of AcH3 in the hippocampus (figure 4b). Suggesting that mice with increased AcH3 in both regions may be generally hypoactive, they were found to be significantly less active, as measured by total distance traveled, than the other two groups of mice on day 1 (figure 4c). Similar results were found when groups were compared across all testing days (not shown).

## Discussion

Here we present a novel technique for high-throughput measurement of total histone H3 acetylation in brain tissue at the single cell level. Demonstrating the utility of our flow cytometry approach, we found that total levels of histone H3 acetylation in the hippocampus were positively correlated with center measures in the open-field paradigm, suggesting that increased total hippocampal AcH3 is related to decreased anxiety-like behavior. A relationship between total levels of histone acetylation and individual variability in behavior suggests future experiments to understand mechanisms through which general levels of histone acetylation may influence brain function. Further, the success of our approach supports the development of additional assays to measure other aspects of global chromatin regulation. Since flow cytometry allows simultaneous detection of multiple antigens in a single cell, we believe our procedure could be expanded to detect several types of epigenetic modifications, allowing for the gathering of correlative data for each cell.

The current study expands on an earlier study where individual differences in open-field exploration were associated with large-scale differences in gene expression levels in the hippocampus of genetically identical mice[12]. Results indicate levels total AcH3 in the hippocampus but not in other brains were related to epigenetic variability in a hippocampus-associated behavior, open-field exploration. The AcH3-related variability in open-field center measures suggests that total AcH3 may be more specifically associated with anxiety. Thus, a more extensive behavioral panel might be useful to follow up on the suggested role of total hippocampal AcH3 in modulating anxiety. Because the hippocampus also influences other behaviors such as learning, it would also be interesting to determine whether variability in total AcH3 is associated with variability in learning-related tasks. This is particularly relevant given studies demonstrating that treatment with histone deacetylase inhibitors can improve learning and memory in a mouse model of Alzheimers[17]. Finally, it may also be interesting to follow up on a possible interaction between total levels of AcH3 in the hippocampus and striatum. Results suggest that high levels of total AcH3 in different brain regions may have variable effects on the same behavioral measures. Larger studies with increased power are needed to validate and determine the extent of potential interactions.

Initial attempts at determining total H3 acetylation involved the Western blot technique. However, the amount of sample required limited the availability of tissue for other assays (e.g. microarray). Western blot results were also technically difficult to reproduce and the procedure was time and labor intensive. In particular, we found that measurements of histone acetylation systematically varied across Western blot gels with center measurements differing by as much as 30% from periphery measurements of the same sample (not shown). Substantial effort was made to correct this problem by using different gels, transfer conditions, and by standardizing AcH3 to total H3 measurements in the same well. While within well standardization seemed like the appropriate solution, the effects of well position were not the same for total H3 and AcH3 and, therefore, within well standardization could not correct for positional differences, which remained around 30% from periphery to center. In contrast to Western blot, the flow cytometry-based technique was amenable to high throughput and was technically reproducible (figure S2). An ability to detect epigenetic modifications in brain cells may be useful for multiple applications in neuroscience. The presence of multiple lasers and filters in flow cytometers, allows for simultaneous detection of various chromatin modifications and neural markers. Additional applications for flow cytometry may include monitoring chromatin responses to treatments and studying variability over the course of development – studies that would greatly benefit from high sample throughput.

The current study also supports a potential reinterpretation of earlier work documenting shifts in total levels of AcH and other chromatin modifications in the context of multiple paradigms[8]–[11], [18]–[29]. While it has been suggested that modulating total levels of chromatin modifications could be important to the control of repetitive DNA elements[30]–[32], the idea that this type of regulation may also play a role in the regulation of brain function and behavior has not gained traction. For the most part, associations of effects with total levels of chromatin modifications have been suggested to stem from effects at specific genes [28], [33]–[36]. An alternative interpretation is that modulation of total levels of hisotone acetyltion and other chromatin marks may be a naturalistic process that influences cellular plasticity – a possibility supported by studies demonstrating increased neuronal plasticity and memory enhancement with HDACi treatment[8], [9], [17], [23], [24], [37]. Importantly, if regulation of total levels of modifications does play a meaningful role in modulating cellular plasticity, it raises the question as to why there have been so many reports suggesting gene specificity to chromatin regulation. This apparent paradox could relate to the hierarchical structure of biological systems[38]–[40]. Genes lower in a hierarchy and further from master regulators at the top will acquire increased noise in expression levels and chromatin modifications that covary with expression levels because of imperfect information transfer through the system. Thus, large-scale changes in hierarchical cellular systems can appear to be changes in a limited number of genes when arbitrary cutoffs for significance and fold-changes are used. Further, methods that detect chromatin modifications in specific promoters first normalize for differences at the total level. Consequently, methods used for evaluating chromatin around specific genes will miss shifts in chromatin modifications at the total level. Thus, it may be that total and gene specific changes in chromatin are both important and may covary. With this in mind we propose that a biological purpose of modulation of total levels of chromatin marks may be to regulate the ease with which (plasticity) gene expression systems can vary along constrained paths, commonly referred to as gene expression programs. Variability in gene expression programs will be accompanied by relative changes in chromatin around the promoters of genes that are part of the programs. In this sense, gene-specific changes in chromatin marks would be the consequence of primary changes in total levels of chromatin marks that facilitate gene expression program progression.

Though future experiments are needed, our demonstration that normal variability in a hippocampus-related behavior correlated with variability in total levels of histone H3 acetylation in the hippocampus supports the possibility that total levels of chromatin marks are important naturalistic modulator of brain function. The tendency to drill down to the level of specific genes may need to be counterbalanced by the current study and numerous additional studies demonstrating that treatments frequently generate behavior-associated variability in total levels of chromatin marks[8]–[11], [20], [21], [23], [24], [37]. Thus, an apparent synchrony between biological and therapeutic mechanisms is encouraging and supports efforts to deliver established therapies that modulate total levels of chromatin marks to brain regions where adjustments could be beneficial, while avoiding areas where chromatin restructuring would be unnecessary or potentially harmful. Such a strategy is well established within oncology, which may be a useful source for future guidance.

## Supporting Information

Figure S1 **Western blot confirmation of flow cytometric measurements of histone H3 acetylation (AcH3).** High and low AcH3 samples were identified with flow cytometry performed on cell culture and triturated brain tissue samples (3 samples per group). The same samples were evaluated with Western blot detection of AcH3 levels. In all cases, results from Western blot agreed with those from flow cytometry.
(PDF)

Figure S2 **Technical replication of flow cytometry measurements.** After performing flow on 20 samples from the hippocampus, samples were stored overnight and re-run. Measurements were highly correlated across flow cytometry runs (Pearson R = 0.95, p<0.0001).
(PDF)

Table S1 **Spearman correlations of open-field behavioral measures across testing days.** Table shows the Spearman-r values for the correlations between five open-field measures (percent distance in the center, total distance in the center, time spent in the center, entries into the center, and total distance travelled) on testing day 1 with behavioral measures from 2 subsequent testing days. Sessions 1 and 3 were conducted in direct light and session 2 was done in the dark to make the testing environment less anxiogenic. Results show there were significant correlations between nearly all measures on subsequent testing days and the behavior of mice on testing day 1. Results indicate that day 1 measurements predict stable individual differences in open-field behavior.
(PDF)

Table S2 **Spearman correlations of open-field behavioral measures with regional measurements of AcH3.** Table shows the Spearman-r values for the correlations between total levels of histone acetylation in 3 brain regions and levels of five open-field measures (percent distance in the center, total distance in the center, time spent in the center, entries into the center, and total distance travelled) across three testing sessions over 28 days. Spearman correlations were used because behavioral measures were often not normally distributed. Sessions 1 and 3 were conducted in direct light and session 2 was done in the dark to make the testing environment less anxiogenic. Results show there were significant correlations of all center measures tested in the light on days 1 and 28 with AcH3 in the hippocampus. There were trends for significance for measures done in the dark with AcH3 in the striatum. Striatal results were mostly explained by four mice with high AcH3 in both the striatum and hippocampus that had very low activity.
(TIFF)
