# Supplementary figures and images for: Total Levels of Hippocampal Histone Acetylation Predict Normal Variability in Mouse Behavior
Source: PLoS One. 2014 May 2;9(5):e94224. doi: 10.1371/journal.pone.0094224 (PMC4008481; doi:10.1371/journal.pone.0094224)

\*  $p < 0.05$   
\*\*  $p < 0.01$

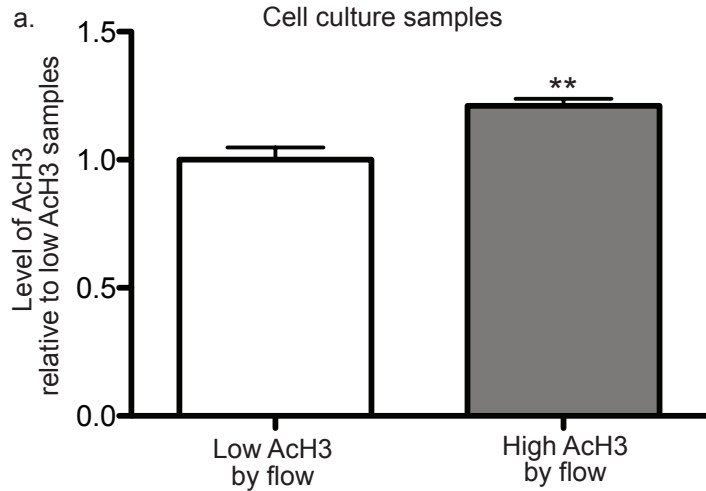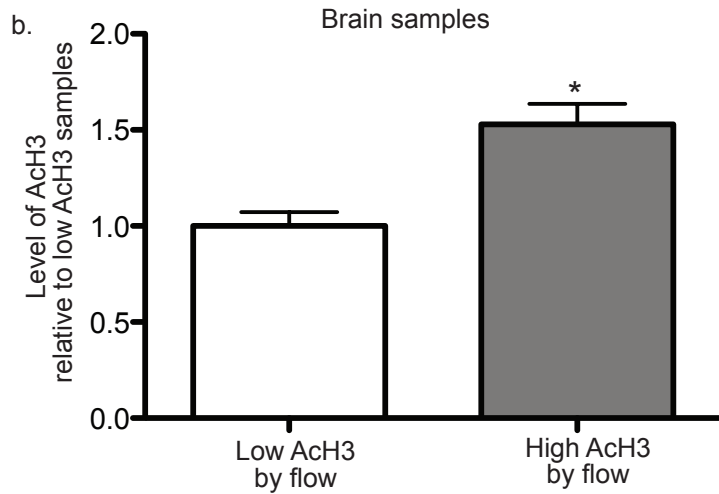

Supplement: Figure S1 — Western blot confirmation of flow cytometric measurements of histone H3 acetylation (AcH3). High and low AcH3 samples were identified with flow cytometry performed on cell culture and triturated brain tissue samples (3 samples per group). The same samples were evaluated with Western blot detection of AcH3 levels. In all cases, results from Western blot agreed with those from flow cytometry. (PDF) [file pone.0094224.s001.pdf]

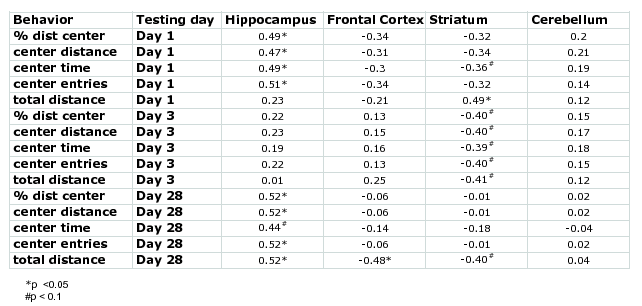

Supplement: Table S2 — Spearman correlations of open-field behavioral measures with regional measurements of AcH3. Table shows the Spearman-r values for the correlations between total levels of histone acetylation in 3 brain regions and levels of five open-field measures (percent distance in the center, total distance in the center, time spent in the center, entries into the center, and total distance travelled) across three testing sessions over 28 days. Spearman correlations were used because behavioral measures were often not normally distributed. Sessions 1 and 3 were conducted in direct light and session 2 was done in the dark to make the testing environment less anxiogenic. Results show there were significant correlations of all center measures tested in the light on days 1 and 28 with AcH3 in the hippocampus. There were trends for significance for measures done in the dark with AcH3 in the striatum. Striatal results were mostly explained by four mice with high AcH3 in both the striatum and hippocampus that had very low activity. (TIFF) [file pone.0094224.s004.tiff]
