# Supplementary material for: Total Levels of Hippocampal Histone Acetylation Predict Normal Variability in Mouse Behavior
Source: PLoS One. 2014 May 2;9(5):e94224. doi: 10.1371/journal.pone.0094224 (PMC4008481; doi:10.1371/journal.pone.0094224)
Supplement: Figure S2 — Technical replication of flow cytometry measurements. After performing flow on 20 samples from the hippocampus, samples were stored overnight and re-run. Measurements were highly correlated across flow cytometry runs (Pearson R = 0.95, p<0.0001). (PDF) [file pone.0094224.s002.pdf]

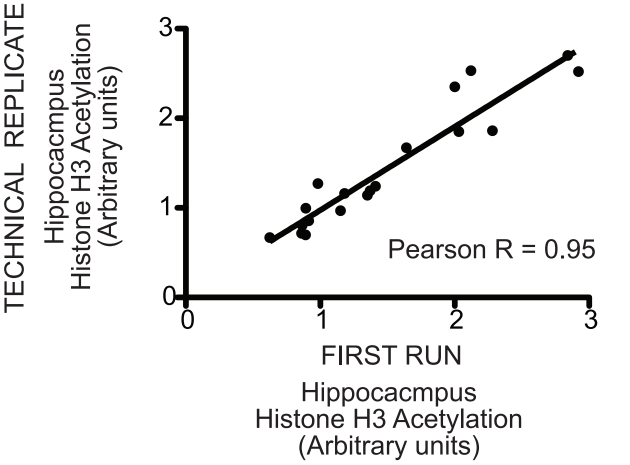
TECHNICAL REPLICATE
Hippocacmpus
Histone H3 Acetylation
(Arbitrary units)
3
2
1
0
Pearson R = 0.95
0
1
2
3
FIRST RUN
Hippocacmpus
Histone H3 Acetylation
(Arbitrary units)
